# Demographic Scenarios of Future Environmental Footprints of Healthy Diets in China

**DOI:** 10.3390/foods9081021

**Published:** 2020-07-30

**Authors:** Aixi Han, Li Chai, Xiawei Liao

**Affiliations:** 1 International College Beijing, China Agricultural University, Beijing 100083, China; 18523157085@163.com; 2 Chinese-Israeli International Center for Research and Training in Agriculture, China Agricultural University, Beijing 100083, China; 3 College of Environmental Sciences and Engineering, Peking University, Beijing 100871, China; 4 School of Environment and Energy, Peking University Shenzhen Graduate School, University Town, Shenzhen 518055, China

**Keywords:** water footprint, carbon footprint, ecological footprint, healthy diet, food consumption, demographic scenarios

## Abstract

Dietary improvement not only benefits human health conditions, but also offers the potential to reduce the human food system’s environmental impact. With the world’s largest population and people’s bourgeoning lifestyle, China’s food system is set to impose increasing pressures on the environment. We evaluated the minimum environmental footprints, including carbon footprint (CF), water footprint (WF) and ecological footprint (EF), of China’s food systems into 2100. The minimum footprints of healthy eating are informative to policymakers when setting the environmental constraints for food systems. The results demonstrate that the minimum CF, WF and EF all increase in the near future and peak around 2030 to 2035, under different population scenarios. After the peak, population decline and aging result in decreasing trends of all environmental footprints until 2100. Considering age-gender specific nutritional needs, the food demands of teenagers in the 14–17 year group require the largest environmental footprints across the three indicators. Moreover, men’s nutritional needs also lead to larger environmental footprints than women’s across all age groups. By 2100, the minimum CF, WF and EF associated with China’s food systems range from 616 to 899 million tons, 654 to 953 km^3^ and 6513 to 9500 billion gm^2^ respectively under different population scenarios. This study builds a bridge between demography and the environmental footprints of diet and demonstrates that the minimum environmental footprints of diet could vary by up to 46% in 2100 under different demographic scenarios. The results suggest to policymakers that setting the environmental constraints of food systems should be integrated with the planning of a future demographic path.

## 1. Introduction

To ensuring food security is commonly recognized as a global challenge, the United Nations has set “Ending hunger, achieving food security and improved nutrition, and promoting sustainable agriculture” as the second of its seventeen Sustainable Development Goals for the year 2030 [1]. By 2050, the world needs to feed nine billion people with food demand being 60% higher than it is today [2]. With the largest population in the world, China’s food security plays an important role in underpinning its social and economic development. To ensure food security, China set a goal of complete self-sufficiency for grains and basic self-sufficiency for meat, eggs, milk, and vegetables as early as 1996 [3].

The growing amount of food production has exacted a heavy toll on the environment and natural resources. The food system accounts for more than 25% of global greenhouse gas (GHG) emissions [4], 70% of the freshwater use, and occupies more than one third of all potential arable land [5,6]. Many previous studies have used the indicators of environmental footprints, including carbon footprint (CF), water footprint (WF), and ecological footprint (EF) to evaluate human food systems’ impacts on the natural environment and resources [7,8,9]. WF, CF, and EF respectively measure the total amount of water consumed, carbon emitted and ecological assets, e.g., land required throughout the life cycle of the final consumption of certain goods and services, e.g., food, of a given population [10,11].

Besides the growing amount, rapid urbanization and people’s bourgeoning lifestyle has led to substantial dietary pattern changes. A general trend of changing from a vegetable-dominated diet to a livestock-dominated diet is widely seen in many developing countries [12,13]. An average Chinese person’s calorie intake from red meat has increased by 16 times from 1961 to 2017 [14]. However, such a change has also brought health concerns such as overweight issues due to high consumption of animal products, which are considered to be the main causes of a number of different non-communicable diseases [15,16].

The environmental implications of such diet pattern changes have gained increasing appreciation as, compared to a vegetable-based diet, animal products require much higher amounts of land and water resources and emit more carbon emissions throughout the life cycle [17,18]. Eighty percent of GHG emissions related to the food system are associated with livestock production [19,20] and the current livestock sector counts for 70% of total agricultural land use [21]. In China, diet pattern transition has already surpassed population growth to be the primary driver of the increasing environmental pressure posed by the food system [22,23].

In order to tackle the negative healthy impacts brought by growing high-livestock food consumption, China updated its nutrition-based dietary reference intake (DRI) guidelines and has issued corresponding Dietary Guidelines in 2016 [24]. Although environmental concerns were not incorporated in the formulation of the above documents, many scholars have shed light on the environmental co-benefits that can be realized through those encouraged diet pattern changes [7,25]. It has been proven that vegetarianism, semi vegetarianism, diets with little meat, Mediterranean diets, etc., can significantly reduce the food systems’ environmental footprints [26]. For example, Aleksandrowicz et al. pointed out that water consumption can be reduced by 50% by adopting a more sustainable diet mode while GHG emissions and land occupation can be reduced by 70–80% [27]. However, it should be noted that most existing studies were carried out based on the standard dietary pattern of adults without considering demographic differences. Song et al. included the impacts of age-gender specified dietary patterns and estimated that dietary changes offer the potential to reduce carbon, water, and ecological footprints of the Chinese food systems by 24%, 15%, and 22% respectively in 2050 [8].

However, while Song et al. applied a single fertility rate based on the United Nations World Population Prospects in their study, China completely abolished its so-called “One-child” policy that was implemented 35 years ago in order to limit most Chinese families to only one child each [8,28]. Since 2015, every couple is allowed to have two children. The “Universal Two-Child” policy implemented in 2016 has directly brought about the birth of second children. According to the Statistical Bulletin on the Development of Health Care in 2017 published by the National Health Commission of PRC, in 2017, there were 17.58 million live births in hospitals nationwide, with more than 50% of children born with two children and above. At the annual meeting of the Chinese Population Association held in July 2017, Peian Wang, deputy director of National Health and Family Planning Commission of PRC (People’s Republic of China), said that since the implementation of the “Universal Two-Child” policy for more than a year, the number of births nationwide has increased although the number of women of childbearing age is declining. In 2016, the national number of new births exceeded 17.5 million, an increase of 950,000 compared to 2015’s 15.65 million births, and an increase of 630,000 compared to 2014’s 16.87 million births [29]. The comprehensive effect of the “Universal Two-child” policy was gradually released, and China’s population structure will change further. Therefore, the impact of future population changes in Chinese society caused by population policies on the environmental impact of the food system deserves further study.

Furthermore, China’s food systems are facing increasing environmental challenges. First, China has committed to cutting its carbon intensity by 40–45% from 2005 levels by 2020 and reaching the peak levels before 2030 [30]; secondly, climate change is expected to aggravate water scarcity in some regions by changing water regimes, both in terms of availability and variability; last but not least, rapid urbanization is shifting an increasing amount of arable land to urban areas, which is set to constrain the available land resources for food production.

The previous studies have proved that adopting a healthier diet is able to significantly reduce environmental footprints. It is informative for policymakers to determine how much environmental pressure would be posed at minimum by food consumption when aiming at human health goals. This minimum environmental pressure can be quantified according to the dietary guidelines; however, this is uncertain and strongly depends on demographic characteristics. In this study, we reveal how the minimum environmental footprint of diet varies with demographic characteristics by integrating the demographic study with the environmental footprint assessment. This study has enlightenments (1) for further researches that demographic characteristics need to be considered when studying the environmental footprints of diet, and (2) for policymakers that demographic characteristics needs to be considered when setting future environmental constraints and redlines for food systems. Results in this study are of global significance because, while China has the world’s largest population to feed, it has also overtaken the US in 2006 as the largest carbon emitter in the world. Maximizing the carbon reduction potentials of China’s food sector will be indispensable to reach global climate change mitigation targets. Furthermore, China is one of the 17 mega-biodiversity countries in the world and harbors nearly 10% of all plant species and 14% of animals on earth [31]. Land and water conservation in China are important for global biodiversity conservation efforts.

## 2. Materials and Methods

### 2.1. Forecasting China’s Population Structure toward 2100

We use PADIS-INT software in this study to predict China’s population size and structure from 2020 to 2100. The PADIS-INT software is developed based on the queue element method, which is a commonly used method to predict the population [32]. We do not consider migration in this study as the overall net migration rate is low in China [10,33].

The PADIS-INT software is developed based on the cohort-component method, which is a commonly used method to predict the population [32]. The cohort-component method looks at the changes in the population of different death, fertility, and migration risks during the forecast period. Simulating this change requires corresponding death, fertility and migration parameters. Here, we do not consider migration in this study as the overall net migration rate is low in China [10,33]. For the age and gender structure of the starting population, which is the most important factor in predicting the overall situation, interpolation techniques are used to make reasonable predictions. PADIS-INT provides a linear interpolation method for assignment in the main age group. That is, given any two points, an interpolation method can be used to calculate and specify the value of the middle year between the two points. The interpolation method follows the following formula:

(1) $$P_{i}^{1}=\frac{1}{5}P_{j}^{5}\cdot \exp\left\{\frac{1}{5}\left(i-a\right)\cdot \ln\left[\left(\frac{1}{5}P_{j+1}^{5}\right)/\left(\frac{1}{5}\right)P_{j}^{5}\right]\right\}=\frac{1}{5}P_{j}^{5}\cdot \left[{\left(P_{j+1}^{5}\right)}^{\frac{1}{5}\left(i-a\right)}/{\left(P_{j}^{5}\right)}^{\frac{1}{5}\left(i-a\right)}\right]$$

where *i* means single-year-old group; *a* represents the age group with a mantissa of 2 or 7 that is slightly less than the age of the single-year group (that is, the middle age group in the five-year group (*i − a*) actually refers to the age difference between the two); *j* represents the five-year-old group where *a* is located; and *P* represents the corresponding population.

When using PADIS-INT to predict the population by age and sex, the key is to set the fertility level, life expectancy, death pattern and sex ratio at birth [33,34]. This article is based on the data of the sixth national census, combined with the analysis of previous census data and the analysis of social and population development trends, the basic parameters are set as follows:

**TFR:** The total fertility rate refers to the average number of children born to each woman during childbearing age in a specific country or region. Although scholars still find a lot of controversy about the actual fertility level, it is generally believed that the total fertility rate is about 1.5–1.6 [10]. In 2016, the “Universal Two-Child” policy was launched, aiming to ease the pressure of aging. This policy has had a profound impact on the Chinese population size and structure since it allows every family to have two children, which may influence the fertility rate of China. Zhai et al. have pointed that the “Universal Two-Child” policy will bring about a relatively significant rebound in China’s TFR and the highest TFR may exceed 1.8 but will not exceed 2 [35]. After the cumulative effect is released, the TFR will be in 1.6–1.7. It was also believed that raising the TFR closer to the intergenerational replacement level of 2.1through policy adjustments, such as 1.77 to 1.94, is the most optimistic assumption [34]. The National Population Development Strategy Research Report proposed that if the total population peak is controlled at about 1.5 billion people, the national total fertility rate should be maintained at about 1.8 over the next 30 years, and a value higher or lower than this number is not conducive to the coordinated development of the population and society [36].

Therefore, we propose three scenarios for setting TFR parameters to predict all possibilities from the influence of the “Universal Two-Child” policy. Before that, we set the TFR of 2018, the starting year, to 1.637 according to WPP (World Population Prospects) [28]. In the first scenario we set the “Universal Two-Child” policy as having not caused changes in fertility levels, so the TFR maintains its current level with slight fluctuations. It fell to 1.5 in 2030, which is the current range of fertility levels, and remained unchanged from then. In the second scenario, we considered that the “Universal Two-Child” policy had some impact on TFR but one that was not so influential. Because the goal of China’s family planning rate is to be stable at 1.8 by 2020, it is reasonable to set the TFR as rising to 1.8 in 2030 and then slowly dropping to 1.6 in 2099 [29]. In the third scenario, we consider that the “Universal Two-Child” policy can significantly increase the Chinese population, so it is assumed that China’s TFR will reach 2.0 in 2030, close to the intergenerational replacement level of 2.1, and this assumption is regarded in the literature as the greatest possibility of China’s total factor productivity [37].

**Life expectancy:** The Population Division of the National Bureau of Statistics points out that in the process of increasing the average life expectancy of China’s population, women have increased faster than men, and the difference between the two has also further expanded. The law of change is consistent [34]. According to the WHO 2019 population life expectancy ranking, the average life expectancy of females in China was 77.6 years in 2018 and 74.6 years for males. We set the life expectancy of men to increase by 1.26 every five years, reaching 82.46 by 2050; the life expectancy of women to increase by 0.17 every year, to reach 85.38 years by 2050.

**Death pattern:** The core element of the population forecasting method based on the cohort element method is to calculate the survival ratio according to age, which needs to be supported by the life table. The basic calculation formula is:

(2) $$N_{t2}\left(x+n\right)=N_{t1}\left(x\right)\cdot \left(L\left(x+n\right)/L\left(x\right)\right)$$

where *N*_*t*1_(*x*) represents the population in age *x* at time *t*1; *N*_*t*2_(*x*+*n*) represents thepopulation in age (*x* + *n*) at time *t*_2_; and *L* is the survival probability in the specific age.

The model life table is abstractly synthesized from the actual life table. As an empirical and standard death mode, it can provide more convenience in constructing the corresponding life table according to the average life expectancy. The Coale-Demeny mortality table was published in 1966 and was built on the basis of 192 actual life tables [38,39]. Among them, the Western model was built on the data of 130 actual life tables, which mainly come from countries in Africa and Asia, such as Israel, Japan, the Taiwan Province of China, and South Africa. There is no obvious systematic deviation in their death data. Therefore, when applied to Chinese population predictions, this group of models is more widely representative than the other groups and is regarded as the standard model life table. So we adopt the Coale-Demeny mortality table in this study to perform age shift and to use is a as death pattern.

**Sex ratio at birth:** The sex ratio at birth refers to the ratio of the total number of male babies born to a total number of female babies in a certain period, usually expressed as the number of male babies per 100 female babies. The “Universal Two-Child” policy is expected to alleviate the imbalance of sex ratio at birth in China. While the data of the sixth census (2010) shows that the sex ratio at birth in China is 118.06, the normal range identified by the United Nations is from 102 to 107 [40].Zhang, a former director of the National Population and Family Planning Commission, pointed out that the implementation of the “Universal Two-Child” policy could alleviate the imbalance in the sex ratio at birth in China, and it shows a downward trend within a certain range [10].Wang pointed out that the “inflection point” of the sex ratio of the birth population in China has emerged and is continuing to strengthen comprehensive governance. Under the conditions, the sex ratio at birth will enter a declining process [41]. Therefore, we assume that the sex ratio will reduce to 107 in 2030 and will remain unchanged after that.

Table 1 summarizes key parameters used in this study to predict China’s future population size and structure until 2100.

### 2.2. Food Systems Environmental Footprint

The environmental footprint coefficients were obtained from a database for the Double Food—Environmental Pyramid (DFEP) model [42]. Table 2 shows these coefficients. The DFEP database has been adopted and works reliably in recent studies assessing the environmental footprint of China’s food system [7,8]. The lifecycle footprint assessment based on this database is in a boundary “from cradle to gate”, i.e., the stages of transportation, storage, and retailing are not included.

Three types of footprints are considered in this study to examine environmental impacts, namely water footprint, carbon footprint, and ecological footprint. The water footprint is an indicator of water use based on consumption. It measures the total water demand for all products and services produced by the residents of that country or region. Three water footprints are considered here, namely green water, blue water, and grey water. Green water refers to the evaporation of water during food production [43]. Blue water refers to surface runoff and groundwater [44]. Grey water is the fresh water needed to dilute sewage [45]. The carbon footprint measures the total amount of greenhouse gas (GHG) caused by an activity (or accumulated in the life cycle of a product) [46]. The Intergovernmental Panel on Climate Change (IPCC) has proposed global warming in terms of CO_2_ equivalents to assess greenhouse gas emissions [47]. The ecological footprint assesses human impact on ecosystems by measuring how much humans today use nature to sustain themselves. It covers all land (including arable land, forests, construction sites, etc.) and converts it into the same unit of measurement, that is, hectares of production land [48].

The scope of this study is focusing on the environmental footprint embodied in the diet rather than the whole food system, so we did not consider the impact of international trade. Importing food indeed can reduce the internal environmental footprint for a country, and therefore the global trade model is encouraged to be integrated into and extend this study in future research. For this study, ignoring the imports is acceptable as China currently has a very high self-sufficient rate of over 85% [36]. This ratio is expected to elevate more in the future, especially after COVID-19.

### 2.3. Deriving Food Consumption Data from Dietary Guidelines

Although the nutrient group item can more accurately assess the quality of the diet, it also lacks operability to a certain extent, so the use of the food group can make the index tool more acceptable and easier to operate. Therefore, we use the food group data given in the dietary guidelines, that is, the recommended daily dietary intake measured by grams per day. We have selected different guide data for different age groups: the 0–5 years-old population adopts Dietary Guidelines for Chinese Women and Children (2016 version); the 6–18 years-old population adopts the Chinese Student Meal Nutrition Standard (WS/T 554-2017), the 18–64 years-old group adopts the Chinese Dietary Guidelines(2016 version);and people over 64 years old adopt the Dietary Guidelines for the Elderly (WS/T 556-2017) [49].All relevant data can be obtained from the website of The Chinese Dietary Guidelines.

At the same time, we divided the food group into 11 categories and unified them with the previous data for environmental footprint: (1) cereals and tubers,(2) vegetables,(3) fruits,(4) red meats and poultry,(5) eggs,(6) aquatic products,(7) dairy products,(8) soybeans and nuts,(9) oils, (10) salt, and (11) sugars.

### 2.4. Environmental Footprints of Age-Gender Specified Food Consumption

**Figure 1:**
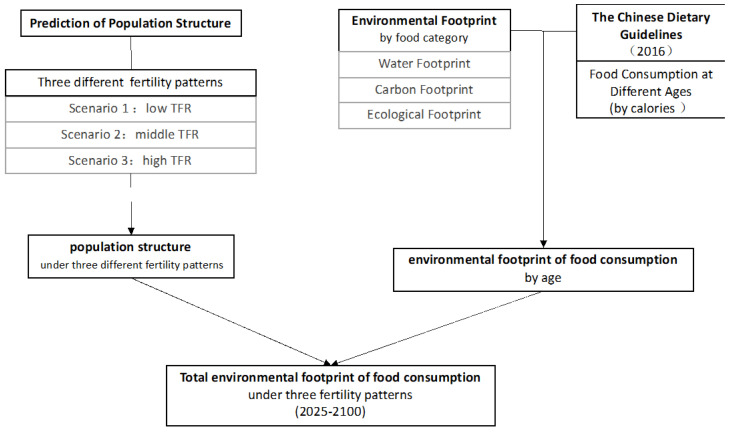
Research framework.

From dietary guidelines, we can get a detailed energy recommended intake (i.e., EER) for each age group and the caloric amount of different food items. The optimal dietary structures of different age groups can be obtained based on such information. Furthermore, based on the environmental footprint coefficients of the consumption of different food items, the environmental footprints of food consumption of people in different age groups can be calculated, which can be used as an indicator of the minimum environmental footprints of food consumption that meet the lowest nutritional requirements of different age groups. Similar to the previous prospective study, we assume that environmental footprint intensities of food consumption will remain the same into the future [50]. Our analyses focus on the impacts of two prospective changes in China’s future food system: (i) the dietary guidelines are used to understand the impacts of future age- and gender-specific dietary patterns; (ii) scenario analyses are conducted to show the impacts of demographic changes. The research route and framework is summarized in Figure 1 below.

## 3. Results

### 3.1. Future Population Size and Structure in China until 2100

**Figure 2:**
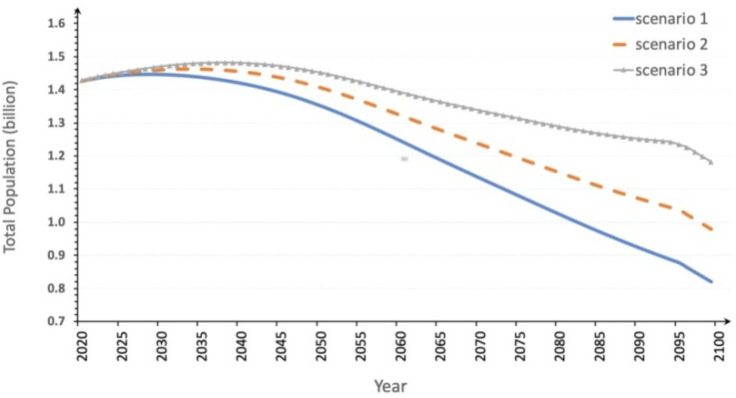
China’s future population until 2100 under different total fertility rate (TFR)scenarios.

Figure 2 shows the changing trend of the total population with time in three scenarios. Under all scenarios, China’s population in the future generally demonstrates a trend of first rising and then declining, with peaks at different times. Under the first scenario with the lowest TFR, China’s population will first grow and reach 1.45 billion in 2028 and will then gradually decline to 1.351 billion and 820 million by 2050 and 2100 respectively. Similarly, the second scenario projects China’s population to grows to 1.46 billion by 2034 and then decline to 1.406 billion and 979 million by 2050 and 2100 respectively. Assuming that the “Universal Two-Child” policy has a significant effect on population growth, the Chinese population is expected to peak at 1.48 billion in 2038 and then gradually decline to 1.45 billion and 1 billion by 2050 and 2100 respectively.

Therefore, although the “Universal Two-Child” policy offers the potential to stimulate population growth in the short term, China’s population is expected to decrease eventually, and people’s willingness to have children is expected not to meet expectations, and the cumulative effect of adjustment and release of fertility policy can only last for a short time period.

**Figure 3:**
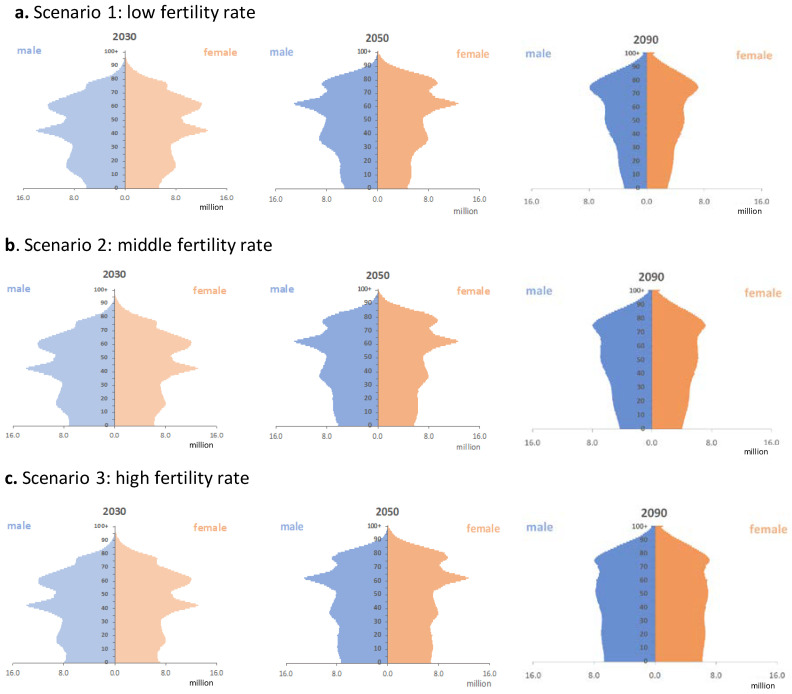
Population pyramids under different scenarios: (**a**) scenario 1 with a low fertility rate; (**b**) scenario 2 with a middle fertility rate; (**c**) scenario 3 with a high fertility rate.

Figure 3 shows that China’s population pyramid shows a slow bottom-shrinking phenomenon. Under the first scenario, the existing fertility level being kept unchanged gradually results in an aging society, while the “Universal Two-Child” policy that is considered in the second and third scenario is expected to alleviate such a trend to different extents.

### 3.2. Age-Gender Specified Food Pattern of the Chinese Population

**Figure 4:**
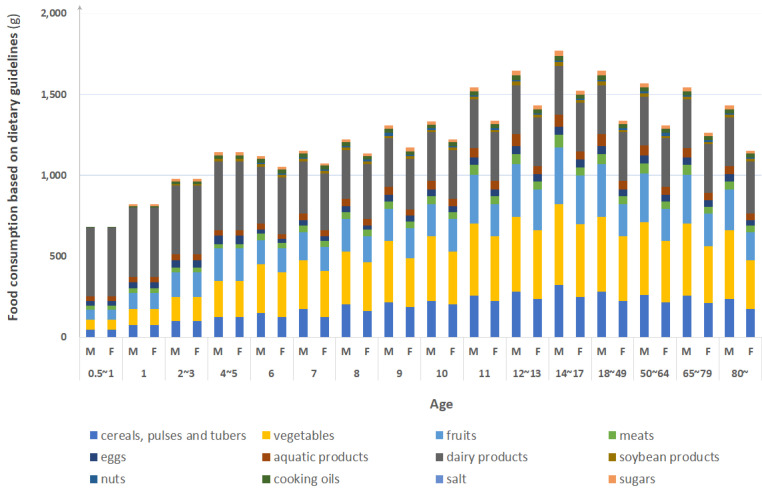
Food consumption by age and gender based on Chinese dietary guidelines.

The optimal dietary patterns that meet the EER by Chinese dietary guidelines of different age groups are demonstrated in Figure 4. It can be seen that the population aged 0–0.5 (six months) mainly depends on breast milk, so there is no specific food consumption. After the age of 0.5, with the growth of age, the total food consumption gradually increases, reaching the peak at the age of 14–17, and then slowly decreases. On the other hand, the difference between the food consumption of men and women begins to emerge after the age of 6, because under normal circumstances, gender differences in food consumption among preschoolers are not considered. Considering gender differences, after the age of 6, men consume a higher amount of food than women in general [51]. In terms of dietary pattern, dairy products and vegetables account for the largest proportions. Take the population of 14 to 17 years for example, dairy products account for about 16.9%, and vegetable consumption accounts for about 28.2%.

### 3.3. Environmental Footprints of Age-Gender Specified Dietaries

Environmental footprint coefficients of different food items used in this study are summarized in Table 2 below. Water Footprint Coefficient (WFC), Carbon Footprint Coefficient (CFC), and Ecological Footprint Coefficient (EFC) respectively represent water consumed, carbon emitted, and land resources used throughout the life cycle of certain food products.

It can be seen from Table 2 that animal products have much higher environmental footprint coefficients than vegetable products; therefore, the increasing consumption of animal products is set to exert increasing pressures on natural resources and the environment.

**Figure 5:**
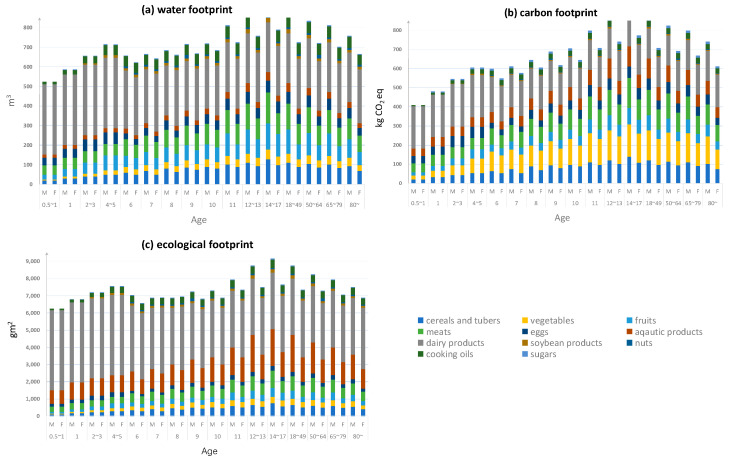
Footprint (**a**), carbon footprint (**b**), and ecological footprint (**c**) of the food system in China.

Figure 5 demonstrates the environmental footprints of age-gender specified dietary requirements according to the national nutritional dietary guidelines. It can be seen that people’s food consumption’s environmental footprints have gradually increased since 0.5-year-olds in general, and people in the 14–17 age group, require the largest environmental footprint to meet their dietary needs. Men require higher environmental footprints for their food requirements than women, with the gap peaking from 14 to 17.

Dairy products, fruits and cereals and tubers require the largest water footprints throughout their life cycles, occupying 45%, 12%, and 11% respectively on average. Dairy products, vegetables and cereals and tubers require the largest carbon footprints throughout their life cycle, occupying 33%, 18%, and 13% respectively on average. In terms of EF, on average, dairy products, aquatic products and meats require the largest EF, occupying 50%, 17%, and 7% respectively on average. It can be seen that dairy products account for the largest environmental footprints, and that the food requirements of the 14~17 age group has the largest environmental footprint. Specifically, fruits, vegetables, and aquatic products, which share in WF, CF, and EF, respectively, all peak at the 14–17 age group, at 16%, 22%, and 22%. Furthermore, food requirements environmental footprints of males have been consistently higher than those of females, with the difference increasing significantly after age 6.

### 3.4. Environmental Footprints for Food Consumption of Chinese Population toward 2100

All three indicators of environmental footprints first increase, then peak around 2030–2035 and then decrease toward 2100. Under scenario 1 where the population trend remains unchanged, WF, CF, and EF first increase and peak in 2029 at 1181 km^3^, 1113 million tons CO_2_eq and 11,809billion gm^2^ respectively. Under scenario 2 and 3 where the “Universal Two-Child” policy effect is to different extents, WF, CF, and EF all peak in a later year, in 2032, at 1190 km^3^, 1120 million tons CO_2_eq and 11,901 billion gm^2^ respectively under scenario 2 where the “Universal Two-Child” policy’s effect is conservative and around 2035 at 1199 km^3^, 1128 million tons CO_2_eq and 12,000 billion gm^2^ under scenario 3 where the “Universal Two-Child” policy’s effects are substantial. After the peaks, dietary pattern change offsets the impacts brought by population increase and leads to environmental footprints decreasing until 2100. By 2100, the minimum CF, WF, and EF associated with China’s food systems range from 616 to 899 million tons, 654 to 953 km^3^ and 6513 to 9500 billion gm^2^ respectively under different population scenarios.

**Figure 6:**
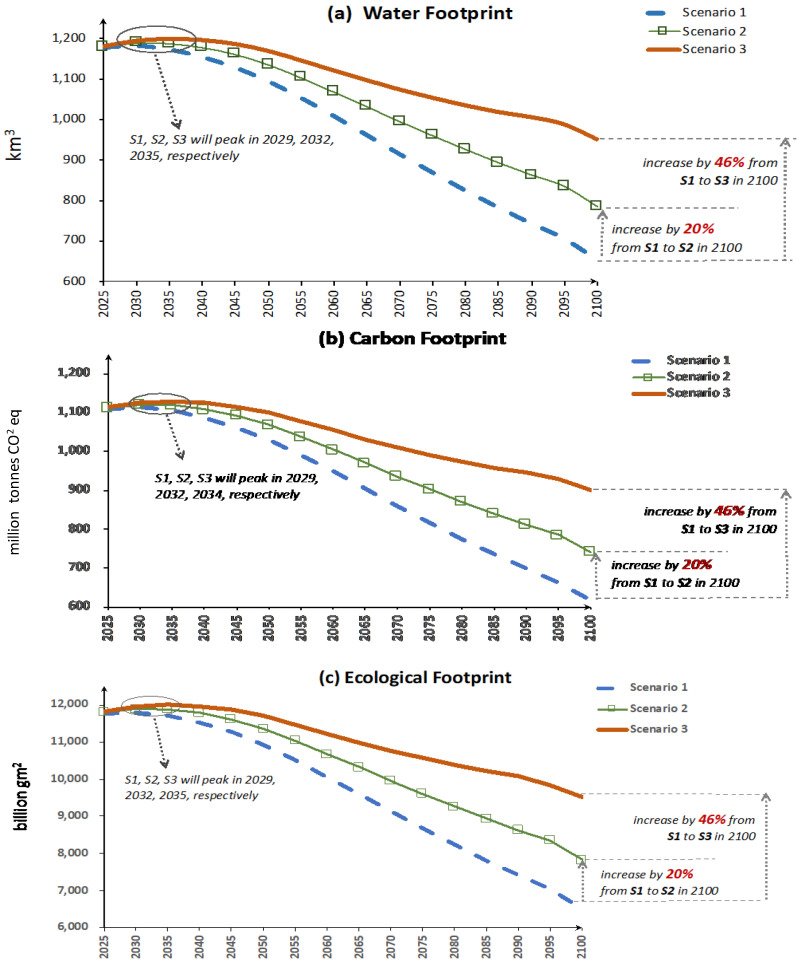
Future trends of food consumption’s total environmental footprint. where (**a**) stands for the trend of water footprint consumption of the food system over time, (**b**) is for the carbon footprint, and (**c**) is for ecological footprint.

It can be seen from Figure 6 that the impacts of the different fertility rates applied in the three population scenarios are negligible until 2030, but gradually become visible from 2050 and are substantial toward 2100.

**Figure 7:**
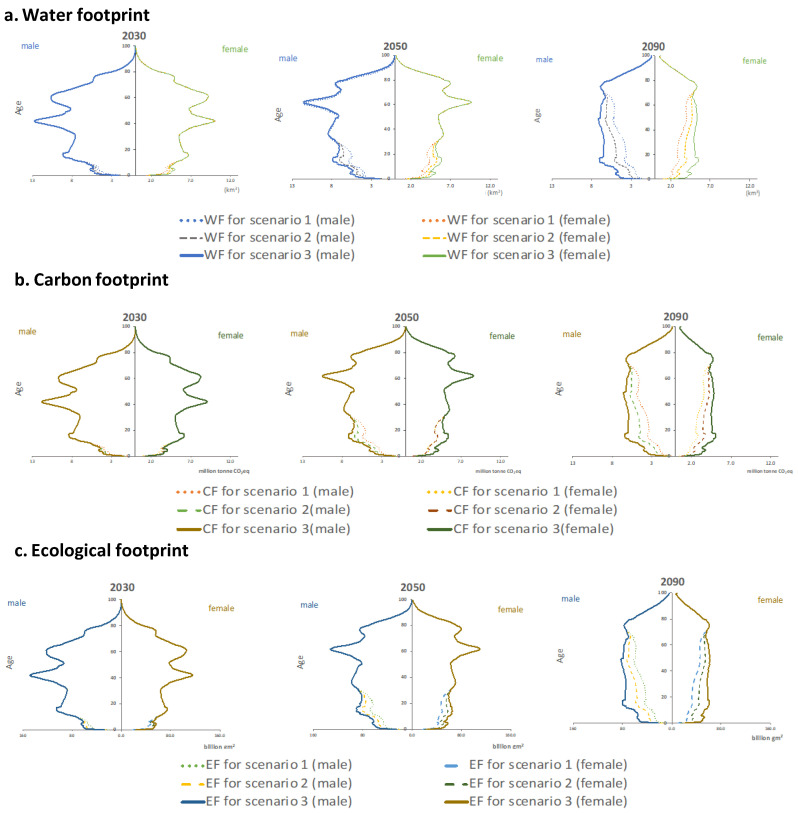
Environmental footprint pyramids demonstrate the changes in the water footprint (**a**), carbon footprint (**b**) and ecological footprint (**c**) under the threepopulation scenarios in 2030, 2050, and 2090.

Two observations worth notice from Figure 7, are that the differences resulted from different population scenarios are most visible in lower aged groups under 2050 and 2100, this is because the “Universal Two-Child” policy of today mainly has impacts on the population size of under 30 by 2050 and under 70 by 2100. Therefore, apparent discrepancies can be observed in environmental footprints of the population under 30 in 2050 and of the population under 70 in 2100. Furthermore, Figure 7 further demonstrates that male members of the society require larger environmental footprints to meet their food demands than their female counterparts since all the figures in Figure 6 have a fatter left part.

## 4. Discussion

### 4.1. Linking the Food System and Its Environmental Footprints

Being constrained by limited planetary boundaries, there is a growing consensus that all anthropogenic activities should take into account and strive to minimize their environmental impacts. With the largest population and unprecedented economic growth, China is facing emergent challenges to meet its demands in a sustainable way. Among all the demands for different commodities, food demand poses the foremost challenge due to its fundamental importance. While food provision processes require large amounts of water consumption, carbon emission and land occupation, China’s food system is posing increasing pressure on its environment and natural resources. In 2009, China pledged to peak its carbon emissions by 2030 in Copenhagen, which is further reiterated in China’s Intended National Determined Contribution. Furthermore, China is also facing stringent finite water and land limitations. China is not abundant in water, with the country’s national average water resources amounting to only one third of the global average at around 1900 m³. Moreover, the scarcity of arable land is a defining feature of Chinese agriculture [52]. In 2015, China fed 18.9 percent of the world’s population with only 8.5 percent of the world’s arable land [36]. Such challenges are further aggravated by the spatially uneven distribution of China’s natural resources. For example, with merely 2 percent of China’s renewable water resources, the Yellow River Basin contributes to 8 percent of the national GDP and supports nearly 9 percent of the national population, 13.3 percent of the arable land. It is therefore of paramount importance to examine the environmental impact of the future food system in exploring a sustainable path meeting both environmental and social objectives. 

According to China’s dietary guidelines, we evaluate the environmental benefits of food pattern change of the Chinese population and demonstrate that while population growth may play the primary role in driving the increasing environmental footprints associated with the food system in the near future, food pattern change offers potentials to offset such impacts and result in net decreases of food system’s environmental impacts after the 2030s.It should be noted that although the guidelines represent the minimal energy and nutrition intakes that are recommended for age and gender-specific groups, the average Chinese person’s current diet has not reached the recommended levels and therefore may have a smaller environmental footprint than our results. However, as recognized by the 2nd SDG (Sustainable Development Goals), nutritious diet plays a fundamental role in sustaining human lives, fostering healthy human capital, and underpinning other aspects of social economic development, results in this study represent the minimal environmental footprints that are required to meet the SDG2 (Sustainable Development Goals) under which scenario every person has access to nutritious food as recommended by the guidelines.

As recognized by previous studies, optimizing food diets helps to contribute to the global climate change mitigation agenda [53,54,55]. China pledged to peak its carbon emissions by 2030, and while its food system makes up 25% of global carbon emissions, our results show that carbon emissions associated with the food system in China will likely peak between 2030 and 2035, which is in line with China’s national efforts to comply with the Paris Agreement [30].

### 4.2. Adopting Age-Gender Specified Food Requirements and Population Scenarios

Different age and gender groups have different dietary needs, therefore using dietary pattern of one single group, normally adults, as a proxy results in overestimation of the food systems’ impacts on the environment. Adopting age-gender specified dietary patterns, results in this study demonstrate that teenagers aged between 14 and 17 require the largest environmental footprint for their food requirements. Dairy products require the largest WF, CF and EF across all age and gender groups. This raises alarms for people’s increasing livestock-based food consumption with a bourgeoning lifestyle. The environmental footprint for food systems could be reduced from both producer and consumer’s sustainable practices. From the producer’s perspective, low environmental-impact farming practices should be encouraged. For example, dripping irrigation is useful in reducing agricultural water consumption. From the consumer’s perspective, environmentally responsible food choices should be promoted. For instance, the increasing consumption of avocado by urbanites is imposing increasing water pressure along the life cycle supply chain. Environmental information disclosure and raising awareness such as via environmental labeling could be a useful way to change people’s consumption behaviors. Furthermore, organic agricultural and husbandry has gained increasing appreciation worldwide, its application in China merits cautions as organic farming require larger amount of water and land inputs as well as greenhouse gas emissions per calorie of food products [56].

While most of the existing studies have examined China’s food systems’ environmental footprints until 2050, it is shown in this study that the population policy’s effects only become significant after that because that’s most new-born become adults whose food requirements reaching the maximum environmental impacts. It is therefore important to look beyond 2050.

### 4.3. Main Contributions and Policy Recommendations

Our results offer three particular novel insights: (1) by adopting different population scenarios, our results have shown that the food system’s environmental footprints may reach the peak several years later delaying from 2030 to 2035 due to population effects resulted from loosening policies. Such effects may cause conflicts with other existing policies, for example, as the food system is a major contributor to the society’s total carbon emissions, the delaying peak of carbon emissions associated with the food system can impede the achievement of China’s carbon emission targets by 2030. While the “Universal Two-Child” policy is expected to generate positive impacts on the shrinking workforce and aging society, its environmental implications need further studying and corresponding policies should be formulated [57]. Enhanced technology advancement should be promoted to reduce the food system’s environmental intensities to mitigate the effects of a growing population. For instance, nitrogen optimization and manure management may be effective in reducing the lifecycle greenhouse gas emission intensity of live-stock consumption [58]. (2) Secondly, our results highlight the importance of adopting a longer-term view in both academic research and policy formation. While most existing studies have examined China’s food systems’ environmental impacts until 2050, our results have shown that the population policy’s effects only become significant after 2050 because that is when most new-born become adults whose food requirements reaching the maximum environmental impacts. While many non-structural environmental policies, such as raising environmental awareness and promoting healthy diet patterns, take decades to be effective, many structural policies, such as irrigation infrastructure, are at risk being locked-in [59].Therefore, it is important for policymakers to look beyond the mid-term and adopt longer term perspectives. (3) Last but not least, by adopting the national age and gender-specific dietary guidelines, our results determine the minimal environmental impacts of the food system in China, which lays the foundation for future studies and for policymakers to evaluate the trade-offs with other sector policies as well as to explore potential synergies.

### 4.4. Limitations and Future Directions

There are certain limitations embodied in this study that merit future studies. First, we estimate the residents’ dietary structures based on the 2016 Chinese Dietary Guidelines. While the current Dietary Guidelines did not take environmental sustainability into consideration, we recommend future dietary guidelines to be further updated upon environmental considerations. Therefore, future studies are recommended to consider optimized dietary structures. Second, water consumption and land occupation have different impacts in different places due to different levels of water and land scarcity. For instance, water consumption in China’s arid northern regions generated higher impacts on water consumption in China’s water-abundant southern regions [60]. Therefore, upon place-specific trade data available, it is meaningful to take into account such considerations to evaluate the lifecycle scarce water and scarce land use by food systems. Food products that are produced from water-abundant and land-abundant regions should be preferred over food produced from where water and land are scarce. Thirdly, environmental-friendly diet modes not only depend on a reasonable diet structure but also on the need to reduce food waste and losses. García-Herrero et al. pointed out that reducing food loss is considered to be an important means to improve food security and reduce the pressure on natural resources [61].

According to research by Chai et al., the dietary patterns of vegetarians, non-vegetarians, and omnivores have different effects on the environment [62]. Therefore, in the following discussion, combined with demographic scenario analysis and different diet patterns, it is more scientific to judge which diet has the least impact on the environment of our planet, so as to make reasonable adjustments to the improvement of the dietary structure. At the same time, China is increasingly playing a significant role in the global food market. Willett et al. have reported that the global food system may have an important impact on human health and environmental sustainability. Providing a healthy diet for the growing global population from a sustainable food system is an urgent challenge [63]. It is therefore recommended to link China’s food system with the global food system in future studies, while combining population scenario analysis with global food allocation, to explore an ideal dietary structure satisfying both health and environmental sustainability.

## 5. Conclusions

We evaluated the minimum environmental footprints, including the carbon footprint (CF), water footprint (WF), and ecological footprint (EF), of China’s food systems into 2100 under different demographic scenarios and using the national age-gender specified energy and nutrition intake requirements. Our results show that food consumption’s environmental impacts peak for the 14–17 age group, the environmental footprint of China’s food system is expected to peak around 2030 to 2035. Under different population scenarios, the peaks of WF, CF, and EF are expected to range from 1181 km^3^, 1113million ton CO_2_eq and 11,809billion gm^2^ respectively to 1199 km^3^, 1128 million tons CO_2_eq and 12,000 billion gm^2^. Higher fertility rates under less strict population policies are also expected to delay those peaks by at most five years. After the peak, environmental footprints of the food system are expected to continuously decline until 2100 and reach 616 to 899 million tons (CF), 654 to 953 km^3^ (WF), and 6513 to 9500 billion gm^2^ (EF) under different population scenarios. The minimum environmental footprints that are required to meet the healthy dietary targets in China are important considerations for policymakers to better coordinate future efforts and to optimize policy portfolios in different sectors, namely, food, demographic, and environmental sectors, in order to realize a path of sustainable development and to achieve the SDGs (Sustainable Development Goals).

## Figures and Tables

**Table 1:** Input parameters of in-scenario analysis.

Parameters		2018	2030	2100
Total fertility rate	Scenario 1	1.637	1.500	1.500
	Scenario 2	1.637	1.800	1.600
	Scenario 3	1.637	2.000	2.000
Life expectancy	Male	74.6	82.46	82.46
	Female	77.6	85.38	85.38
Death patterns		Coale-Demeny mortality table		
Sex ratio		118. 06	107.00	107.00

**Table 2:** Coefficients of the water footprint (WFC), carbon footprint (CFC), and ecological footprint (EFC) of each food category.

Food Item	WFC (m^3^kg^−1^)	CFC (kg CO_2_ekg^−1^)	EFC (gm^2^kg^−1^)
wheat	1.62	0.94	10.63
rice	1.50	2.51	7.80
maize	1.05	0.66	7.50
other cereals	1.50	1.33	8.76
tubers	0.56	0.18	3.00
other legumes	2.44	1.00	21.50
soybean products	2.44	1.00	21.50
nuts	2.44	1.00	21.50
vegetables	0.27	0.93	2.10
fruits	1.05	0.67	4.05
dairy	2.32	1.43	30.00
eggs	3.28	3.23	14.41
beef	15.41	21.36	112.63
lamb	5.26	10.44	76.00
pork	5.99	4.19	24.58
poultry	4.33	3.41	24.50
aquatic products	1.63	3.85	78.25
cooking oils	6.25	2.97	43.97
sugars	0.52	1.35	4.57
